# Metallic nano-structures for polarization-independent multi-spectral filters

**DOI:** 10.1186/1556-276X-6-394

**Published:** 2011-05-23

**Authors:** Yongan Tang, Branislav Vlahovic, David Jones Brady

**Affiliations:** 1 The Department of Physics, North Carolina Central University, Durham, NC, 27707, USA; 2 Department of Electrical and Computer Engineering, Duke University, Durham, NC, 27708, USA

## Abstract

Cross-shaped-hole arrays (CSHAs) are selected for diminishing the polarization-dependent transmission differences of incident plane waves. We investigate the light transmission spectrum of the CSHAs in a thin gold film over a wide range of features. It is observed that two well-separated and high transmission efficiency peaks could be obtained by designing the parameters in the CSHAs for both p-polarized and s-polarized waves; and a nice transmission band-pass is also observed by specific parameters of a CSHA too. It implicates the possibility to obtain a desired polarization-independent transmission spectrum from the CSHAs by designing their parameters. These findings provide potential applications of the metallic nano-structures in optical filters, optical band-pass, optical imaging, optical sensing, and biosensors.

## Introduction

The extraordinary light transmission (ELT) of sub-wavelength-hole arrays in thin metal films are of interest to many researchers, due to their many applications in optical imaging, optical communication, and optical sensing. The fraction of light transmitted through a periodic array of sub-wavelength-hole in a metallic film exceeds the open fraction occupied by the holes at certain wavelengths are related to the periodicity of the hole array [1], and the transmission can be anomalously large as compared to well-established predictions for isolated holes [2]. This ELT phenomenon is due to the localized surface plasmon polaritons (SPPs) [3] of the metal film. Surface plasmons (SPs) are electromagnetic waves propagate along the surface of a metal; they are trapped on the surface due to the interaction between the light wave and the free electrons of the metal; the free electrons respond collectively by oscillating in resonance with the light wave. This resonant interaction constitutes the SP and gives rise to its exceptional properties: the momentum of the SP mode, *ħk*_SP_, is greater than that of a free-space photon of the same frequency, *ħk*_0_, where *k*_0 _= *ω*/*c *is the free-space wave-vector. The frequency-dependent SP wave-vector, *k*_SP _is described as,(1)

where *ε*_d _and *ε*_m _are the permittivities of the dielectric material and the metal, respectively. For *ε*_d _= 1 (air as the dielectric) and a negative *ε*_m _for a metal, it means that the SP wave-vector *k*_SP _is larger than the incoming free-space wave-vector *k*_0_. Therefore, in order to induce the SPs on a metal surface, it is required to compensate the increased momentum. A few techniques are reported to increase the momentum, such that scattering from a topological defect on the surface, such as a sub-wavelength hole, which provides a convenient way to generate SPs locally [4]; the prism coupling to enhance the momentum of the incident light [3,5], and the use of a periodic corrugation in the metal's surface [6]. The surface plasmon resonance is also observed from metal nanocrystal [7], and from bimetallic interface in Au-Ag core-shell structure nanowires [8]. In this article we will discuss the technique of inducing SP with sub-wavelength-hole array, and specially a polarization-independent cross-shaped-hole array (CSHA) for multi-spectral filters and band-passes.

The mechanism model of the light propagating through these sub-wavelength-hole arrays can be described as three steps below. Firstly, the impinge plane wave is trapped on the metal film as localized surface plasmon (LSP) [9-11] by the sub-wavelength-holes; secondly, these localized SP polaritons propagate in the sub-wavelength holes; the process of SPs propagating in these sub-wavelength holes is not as simple as pass through them, in fact, partial of SPs are reflected back to the hole at the exit surfaces, due to the refractive index difference of the hole and that of the two surfaces of the metal film, therefore, the two surfaces of the metal can be viewed as two reflectors of the holes, as a result, the sub-wavelength-holes of the metal film form Fabry-Perot like cavities; Finally, light emits from the other side of Fabry-Perot like cavity (or the metal film). It is known that the shape of the sub-wavelength-hole and the periodicity of the array are the most important two elements in controlling the light in these metallic structures [12]. For a rectangular hole array [13], the transmission efficiency is dependent on the polarization of the incident wave, e.g., it is expected to obtain high transmission efficiency when the polarization is parallel to the short ridge of the hole. In this article, we investigate the optical transmissions as functions of these two elements and the thickness of thin metal films in a CSHA, which is designed to obtain the polarization independent multi-spectral filters and band-passes. As described in the mechanism model above, the transmission spectrum can be managed in a degree by the periodicity and the features of the holes; it shows that it is possible to obtain multi-spectral filters or frequency selective surface (FSS) by manipulate the thickness of the metal film and other parameters of the nano-structures.

### Lorentz-Drude model for dielectric function of gold

It has been shown [14] that the complex dielectric function *ε*(*ω*) of a metal can be expressed in the following form:(2)

where *ε*_1_(*ω*) is the intraband part of the dielectric function and *ε*_2_(*ω*) is the interband part of the dielectric function. The intraband part *ε*_1_(*ω*) is described by the well-known free-electron or Drude model:(3)

where *σ*_0 _is the strength, *ω*_p _is the surface plasmon frequency, and *1/Γ*_0 _is the lifetime (or relaxation). The interband part *ε*_2_(*ω*) is described by the simple semiquantum model resembling the Lorentz result for insulators:(4)

**Figure 1:**
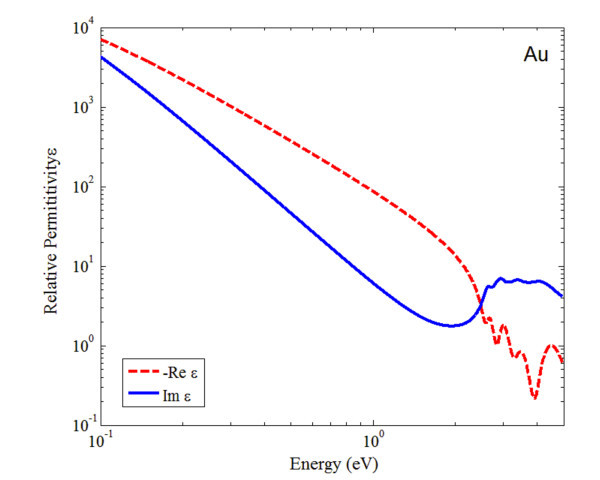
**The plot of the dielectric constant of gold as a function of photon energy**. The red dash line is the negative of the real part of permittivity, and the blue solid line is the imaginary part of permittivity.

Combine Equations 3 and 4, setting *ω*_*j *= 0 _= 0, and rewrite Equation 2, the Lorentz-Drude critical points model for metal permittivity can be described:(5)

where *ω*_p _is the plasma frequency (for gold *ω*_p _= 9.03 eV or 137 nm). The critical point parameters (shown in Table 1) of gold film dielectric function are converted to the finite difference time domain (FDTD) software of MEEP [15] with setting ε_Gf _= 5.135. The plot of the dielectric function of gold (shown in Figure 1) matches with experimental results very well.

**Table 1:** Parameters of gold dielectric function for simulation

***ω***_***j***_	***σ***_***j***_	***Γ***_***j***_
10^-10^	6.2634 × 10^21^	0.0486

2.1201	0.1906	0.1770

2.3525	0.4508	0.3012

2.7116	0.6653	0.5780

3.3530	1.5974	1.1844

4.4214	0.9835	1.9662

### Polarization-independent multi-spectral filters

**Figure 2:**
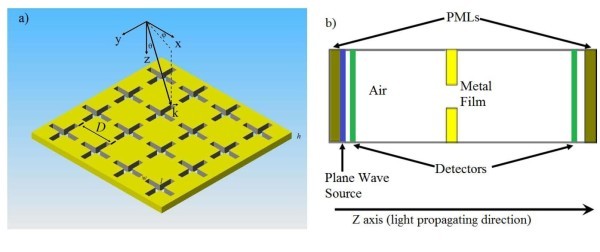
**(a) Sketch of the structure of the array, and (b) sketch (cross-section view) of the unit cell for simulations**.

The optical transmission of a square CSHA as a multi-spectral filter or a FSS is investigated. The array, which is patterned on a metal (gold) film, is shown in Figure 2, where *D *is the period of the array, *w *and *l *are the width of the short side and the length of the long side of the cross-shaped-hole, respectively; *h *is the thickness of the metal film, and its optical properties of the metal are described by the dispersive complex dielectric function above. The software of the FDTD method, MEEP, is applied for the simulations. The FDTD method divides space into discrete grid and then the electromagnetic field evolved in time by using discrete steps. The finer the grid and time steps are, the closer to the true equations of the simulation will be, therefore the better simulation results one could expect.

A plane wave impinges on the CSHA with an incident angle of *θ *(shown in Figure 2). The orientation of the incidence plane is located by the azimuthally angle measured from *x*-axis. When we set *θ *= 0 and *φ *= 0, the plane wave is normal to the metal film. Since the optical transmission is greatly dependent on the polarization of the electric field of the plane wave for rectangular hole arrays, the CSHA is introduced to reduce the dependence of the polarization due to its symmetrical structure for both TE and TM waves. In these simulations, the gold dielectric parameters in Table 1 is applied, and the gold film is sitting in the middle of a computing unit cell (see Figure 2b), and the boundary conditions of the unit cell are set as periodically (Bloch-periodic in both *x *and *y *directions), two perfect match layers (PMLs) are put at both ends (*z *direction) in the unit cell, after the PML, a plane wave source is set to illuminate normally to the metal structures (at *θ *= 0 and *φ *= *0*), and a detectors is placed in front of the PML of the unit cell to measure the transmission spectra by computing the fluxes of these Fourier-transformed electric fields. The thicknesses of the PMLs are dependent on the working wavelength. Therefore, it is important to setup proper thickness of the PMLs to reduce numerical reflection. As mentioned above that the finer the steps the better resolution to be obtained, it is also very important to set a high resolution for the simulations.

**Figure 3:**
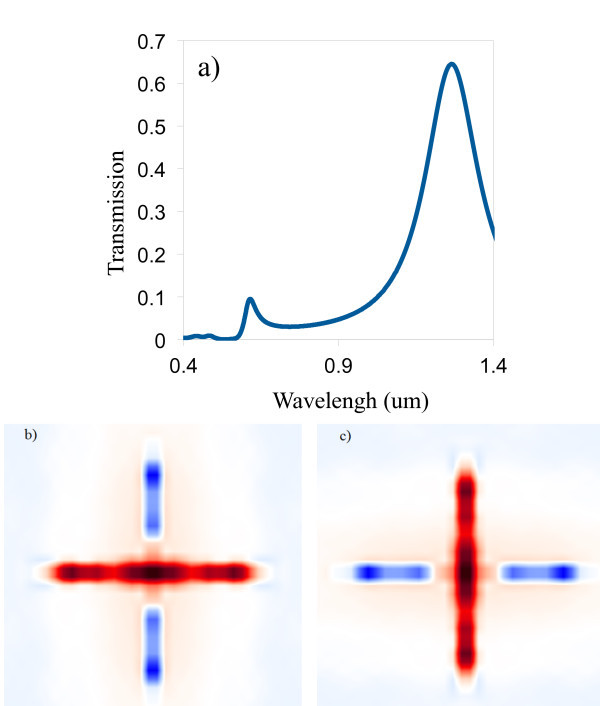
**(a) Transmission spectrum of CSHA (*L *= 300 nm, *W *= 20 nm, period *D *= 350 nm, and *h *= 100 nm); (b) the electric field in the metal film of the TM wave; (c) the electric field in the metal film of the TE wave**.

The metallic CSHAs are illuminated by a p-polarization (TM) monochromatic electromagnetic plane wave (electric field in the plane of the incidence), and an s-polarization (TE) monochromatic electromagnetic plane wave (electric field perpendicular to the plane of incidence), respectively. The simulations show that the TE and TM waves have same transmission spectrum (see Figure 3a) for the CSHAs. However, there is a difference in the electric field distributions of the TE and TM waves in the CSHA: more light is trapped in the horizontal rectangle open (hole) of the cross-shaped hole for TM wave (see Figure 3b), and more light is trapped in the vertical rectangle open of the cross-shaped hole for TE wave (see Figure 3c). These electric field distribution results verify that the efficiency of light coupling to SP is strongly polarization dependent; the shorter ridge of the hole has higher efficiency to induce SPs when the polarization of the light is parallel to it. Therefore, the CSHA is a good candidate for the polarization independent multi-spectral filters or FSS, since the CSHA provides a symmetrical structure for the TE and TM waves.

The simulation results in Figure 3 are obtained by illuminating a plane wave on a CSHA with a period of 350 nm, length of 300 nm, width of 20 nm, and thickness of 100 nm in a gold film. In these cross-shaped-holes, the open area of the cross-shaped hole is about 2 × 300 × 20 nm^2^, which is about 1/10 of the area of a unit cell, which is 350 × 350 nm^2^. The transmission efficiency is about 70% at the center wavelength of 1.25 μm for this CSHA. A range of parameters for the cross-shaped hole (include the length, width, and both of them of the rectangle of the holes) are investigated, the results show that a very similar transmission peak can be obtained by varying these parameters of the CSHAs, though the wavelength center, the band width, and even the magnitude of the transmission peak varies a little bit for the different parameters. These results are consistent with the results that we obtained in the rectangle-hole arrays: the transmission resonance wavelength shifts toward red, at the same time the transmission peak narrows down with same magnitude as the width of the hole decreases; therefore, the width of the rectangular hole is more important on prediction of the transmission peak location; Meanwhile, the length of the rectangular hole plays a more important role on the transmission efficiency and bandwidth. The periodicity of the array as another important parameter is also investigated, the transmission peaks are red shifted from their periodicity, and this result is similar as the transmission peaks' behavior of the rectangle-hole arrays, in other words, for a larger periodicity, a longer wavelength of the transmission peak is observed. Therefore, periodicity of the array and the width of the rectangular hole are two factors in predicting the transmission peak's location.

**Figure 4:**
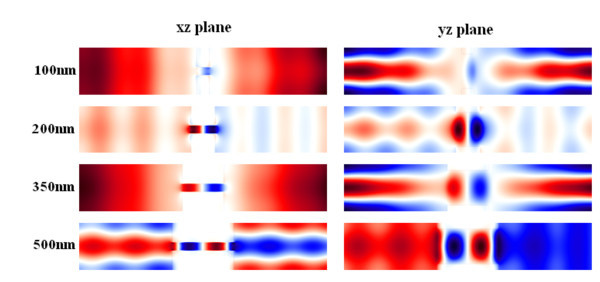
**The cross-section views of *xz *plane and *yz *plane of the plane wave electric field when it is propagating through the sub-wavelength hole arrays of 350 nm rectangular hole (250 nm × 50 nm) array on various thicknesses (100 to 500 nm) of gold film on corresponding thicknesses**.

As we mentioned above, the localized SPs by the sub-wavelength holes propagate on the metal surface and into the holes, then these SPs or electric field experience the difference of the refractive index at the exits of the holes, some SPs emit from the holes and couple to light, and some SPs are reflected back to the holes, as a result, Fabry-Perot kind of cavities are formed due to the difference of the refractive index at the exits of the holes. The role of the Fabry-Perot cavity of these sub-wavelength-hole arrays is important to the applications of these metallic structures as a multi-spectral filter or a FSS. This Fabry-Perot cavity effect is shown by electric field distributions in *xz *plane and in *yz *plane in Figure 4, which is obtained by varying the thickness of the metal film (from 100 to 500 nm) analog to the cavity length, while keeping the periodicity of *D *= 350 nm and the feature of the rectangular hole of 250 nm × 50 nm as constants.

**Figure 5:**
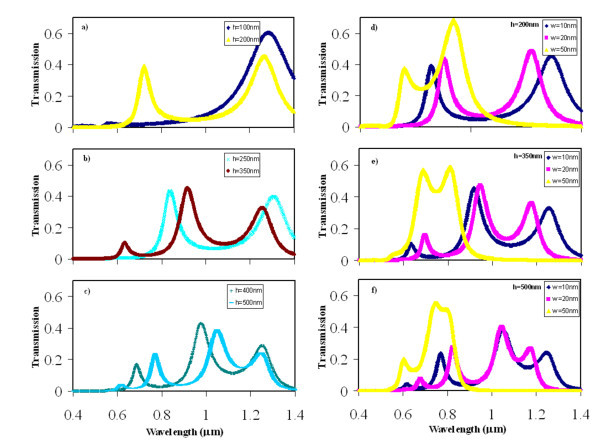
**The transmission spectrum of a CSHA**. (*L *= 280 nm and *D *= 350 nm) with: **(a) **thickness of *h *= 100 nm and *h *= 200 nm with *W *= 10 nm; **(b) **thickness of *h *= 250 nm and *h *= 350 nm with *W *= 10 nm; **(c) **thickness of *h *= 400 nm and *h *= 500 nm with *W *= 10 nm; **(d) ***W *= 10 nm (*L *= 330 nm), 20 nm, and 50 nm and thickness of *h *= 200 nm; **(e) ***W *= 10 nm (*L *= 330 nm), 20 nm, and 50 nm and thickness of *h *= 350 nm; **(f) ***W *= 10 nm (*L *= 330 nm), 20 nm, and 50 nm and thickness of *h *= 500 nm.

The transmission spectra of the Fabry-Perot cavity of the CSHAs are shown in Figure 5. The Fabry-Perot like cavity effect of the thickness of the CSHAs is similar to that of the rectangle-hole array in Figure 4. Widths of *W *= 10 nm, lengths of *L *= 280 nm, and period of *D *= 350 nm are the parameters of the cross-shaped holes used for the plots of Figure 5a,b,c. It is observed that one transmission peak is centered at wavelength of approximately 1.25 μm (Figure 5a) for a thin gold film (*h *= 100 nm); two quite even transmission peaks are observed in Figure 5a,b both in thickness of *h *= 200 nm and *h *= 250 nm, respectively; however, the locations of these peaks are different corresponding the gold film thickness, and the two transmission peaks of 250 nm thick film are closer than these of 200 nm thick film; the third peak in the transmission spectrum emerges for a thickness of 350 nm gold film (Figure 5b); it is also observed that the third transmission peak of the 500 nm thick gold film is higher than that of 400 nm thick gold film (Figure 5c), but the center wavelengths of these three peaks are closer for the 500 nm thick gold film.

Since the length of the hole has more effect on the magnitude of the transmission peak, and the width of the hole has more effect on the location of the transmission peak, therefore, in this investigation, we vary the width of the hole from *W *= 10 nm to *W *= 50 nm, and keep the length of the hole as a constant (*L *= 280 nm). The simulation results of the CSHA with same period and lengths but different widths are shown in Figure 5d,e,f. The plots of Figure 5d show that the CSHAs, with a thickness of *h *= 200 nm, length of *L *= 330 nm and widths of *W *= 10 nm, and length of *L *= 280 nm and widths of *W *= 20 nm and 50 nm in a periodicity of *D *= 350 nm, produce two transmission peaks in the spectrum; these two transmission peaks have same magnitude and are well separated for *W *= 20 nm; The simulations also show that the width of *W *= 50 nm is a little too wide to produce two well-separated transmission peaks (Figure 5d); however, it can produce a broad band pass transmission peak (Figure 5e) with the thickness of 350 nm; the third and fourth transmission peaks emerge in the thickness of *h *= 350 nm and *h *= 500 nm, respectively, for the width of *W *= 10 nm and 20 nm (Figure 5e,f). In conclusion, it is possible to obtain two well-separated and high transmission peaks by design these parameters in the CSHA (*L *= 280 nm, *W *= 10 or 20 nm, *D *= 350 nm, and *h *= 250 nm); it is also possible to produce a high efficiency broad band-pass with the CSHA (*L *= 280 nm, *W *= 50 nm, *D *= 350 nm, and *h *= 350 nm) in a gold film.

**Figure 6:**
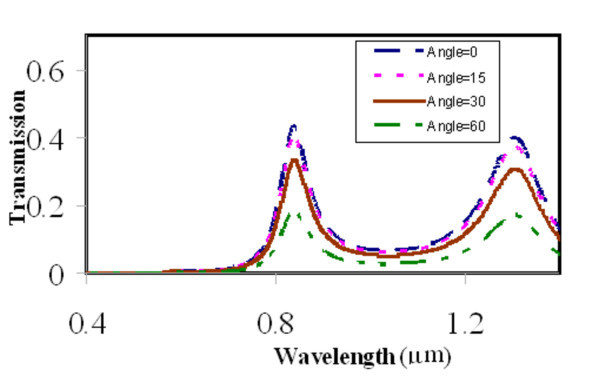
**The transmission spectrum of a CSHA**. (*L *= 330 nm, *W *= 10 nm, *h *= 250 nm, and *D *= 350 nm) with different incident angles of 0, 15, 30, and 60.

It is very important for the CSHAs to have a large incident angle acceptance in the application in the spectral filter field. We vary the incident angle of the plane wave from 0 to 60 in the simulations (*L *= 330 nm, *W *= 10 nm, *D *= 350 nm, and *h *= 250 nm), these results show that the transmission peaks will stay at the same wavelengths, but the magnitudes of the peaks will decrease slightly for larger angles (see Figure 6), and this slightly decrease is acceptable in most of applications.

## Discussion and conclusions

We investigate the process of light propagating in nano-structures of CSHA in gold film, following the localized SPs coupled to light from the hole of the metal film; the SPs experience Fabry-Perot like cavity effect from the metallic structures due to the refractive index at the exits forming reflectors. This Fabry-perot cavity effect in the SPs propagating in the nano-holes can be utilized to produce multi-transmission peaks or broad-band transmission peak. Our simulations show that a CSHA can be a polarization independent multi-spectral filter or a FSS; the increase of the thickness of the metal film leads to multi-modes of light come out from the metallic structures. Two well-separated transmission peaks with same magnitude and a broad band-pass transmission peak are obtained by engineering the parameters of the metallic nano-structures; moreover, these CSHA structures can produce very similar two transmission peaks when the plane wave is illuminating the CSHA with an angle (θ = 0-30). These results indicate that the possibility of the metallic nano-structures in applications of optical communication, optical imaging, optical sensing, and biosensors, etc. The investigations are carried out in the near infrared region. However, our further simulations show that these conclusions also stand in the visible, far infrared, or microwave regions.

## Abbreviations

CSHAs: cross-shaped-hole arrays; ELT: extraordinary light transmission; FSS: frequency selective surface; FDTD: finite difference time domain; PMLs: perfect match layers; SPPs: surface plasmon polaritons; SPs: surface plasmons.

## Competing interests

The authors declare that they have no competing interests.

## Authors' contributions

YT: designed the structures, did the simulations, and drafted the manuscript; BV: participated the structure design; DB: proposed the structures and plotted the applications. All authors read and approved the final manuscript.
